# Genetic Analysis of the Major Capsid Protein of the Archaeal Fusellovirus SSV1: Mutational Flexibility and Conformational Change

**DOI:** 10.3390/genes8120373

**Published:** 2017-12-08

**Authors:** Eric A. Iverson, David A. Goodman, Madeline E. Gorchels, Kenneth M. Stedman

**Affiliations:** Center for Life in Extreme Environments, Biology Department, Portland State University, P.O. Box 751, Portland, OR 97207-0751, USA; ericiverson12@yahoo.com (E.A.I.); dag2@pdx.edu (D.A.G.); mgorchel@wellesley.edu (M.E.G.)

**Keywords:** proteolysis, site-directed mutagenesis, complementation, mutants, morphology

## Abstract

Viruses with spindle or lemon-shaped virions are rare in the world of viruses, but are common in viruses of archaeal extremophiles, possibly due to the extreme conditions in which they thrive. However, the structural and genetic basis for the unique spindle shape is unknown. The best-studied spindle-shaped virus, Sulfolobus Spindle-shaped Virus 1 (SSV1), is composed mostly of the major capsid protein VP1. Similar to many other viruses, proteolytic cleavage of VP1 is thought to be critical for virion formation. Unlike half of the genes in SSV1, including the minor capsid protein gene *VP3*, the *VP1* gene does not tolerate deletion or transposon insertion. To determine the role of the *VP1* gene and its proteolysis for virus function, we developed techniques for site-directed mutagenesis of the SSV1 genome and complemented deletion mutants with *VP1* genes from other SSVs. By analyzing these mutants, we demonstrate that the N-terminus of the VP1 protein is required, but the N-terminus, or entire SSV1 VP1 protein, can be exchanged with VP1s from other SSVs. However, the conserved glutamate at the cleavage site is not essential for infectivity. Interestingly, viruses containing point mutations at this position generate mostly abnormal virions.

## 1. Introduction

Viruses with spindle-shaped virions are rare in the virus world except in viruses infecting archaea from extreme environments [1]. Moreover, very little is known about how these unique structures are formed. How the spindle-shaped viruses that infect thermoacidophilic archaea retain their integrity in the extreme (80 °C) temperatures and low pH (=3) of their environment is also not clear. To answer these questions, we work with model spindle-shaped viruses. These are the fuselloviruses or Sulfolobus Spindle-shaped Viruses (SSVs) [2]. The best-studied of the fuselloviruses is SSV1, originally isolated from *Sulfolobus shibatae* from an acidic hot spring in Beppu, Oita, Japan in the early 1980s [3,4]. SSV1 has a 15,465 bp double stranded circular DNA genome, packaged as positively supercoiled DNA in a spindle or lemon-shaped virion with a short tail at one end [3,5,6]. SSV1 infects a number of *Sulfolobus* species and the virus genome is integrated into the host genome on infection [7,8,9]. How the virus genome is released or how SSV1 binds to *Sulfolobus* is not clear. SSV1 does not lyse cells when produced, yet slows host cell growth [7]. SSV1 virions seem to form by budding through the *Sulfolobus* membrane [10]. Purified SSV1 virions contain virus proteins (VP) VP1 and VP3, hydrophobic capsid proteins, VP2, a DNA-binding protein, VP4, the putative tail protein and host-derived lipids [11,12]. Recently, an approximately 32Å resolution cryo-EM structure for SSV1 was determined and a novel fused-fullerene cone model for the structure was proposed [13]. This model is intriguing, as it is fundamentally geometrically similar to the capsid of human immunodeficiency virus-1 (HIV-1) [14], but not similar at all in sequence. At current resolution it is hard to determine if this model is correct. Even more recently, a comprehensive mutagenesis study of the SSV1 genome was performed, indicating that approximately 1/2 of the 35 Open Reading Frames (ORFs) in the SSV1 genome are not essential for virus function, including the minor capsid protein VP3 [15]. Interestingly, virions lacking the *vp3* gene have aberrant shapes yet still fit the fused fullerene cone model. Not surprisingly, the major capsid protein gene, *VP1*, was shown to be essential for virus function [15].

**Figure 1:**
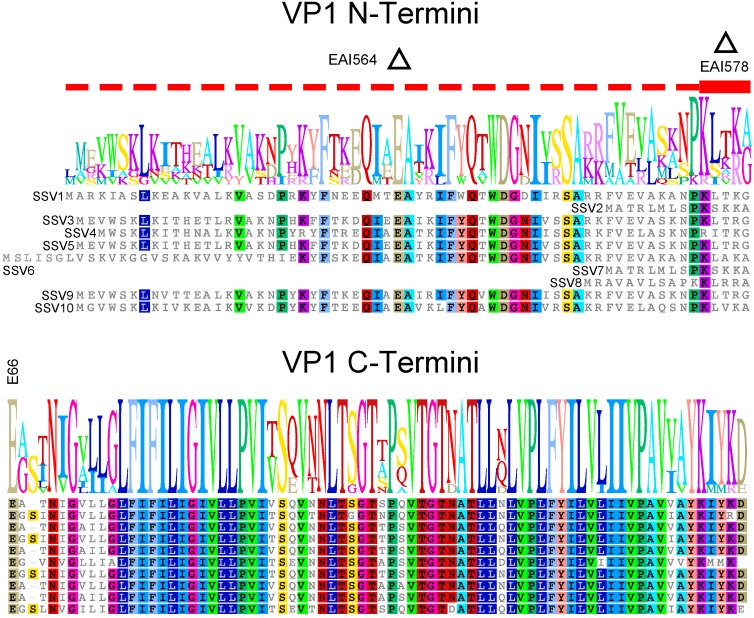
Amino Acid Sequence Alignment of Fusellovirus VP1 Proteins. VP1 protein sequences from Sulfolobus Spindle-shaped Virus 1 (SSV1) (**top**) to SSV10 (**bottom**) were aligned using Geneious V. 8.2 (Biomatters, Auckland, New Zealand) and edited by hand. Accession numbers are listed in reference 15. Identical amino acids are highlighted in color. Sequences present in 9/10 sequences or as an insert are also highlighted in color. A sequence logo is shown above the alignment. The alignment is split at the universally conserved glutamate (E) at position 66 in the SSV1 sequence where proteolysis occurs and labeled. The 6 amino acids that are deleted in construct EAI578 are indicated with a red bar and a delta symbol. The sequence that is deleted in construct EAI564 is indicated with a dotted red line and a delta symbol.

The SSV1 VP1 protein appears to be made as a precursor. In purified SSV1 particles, the N-terminal residue of the VP1 major capsid protein is a glutamate, indicating that the protein is made as a precursor protein and is then proteolytically cleaved by an unknown protease [5,11,12]. Proteolytic cleavage of virus structural proteins is very well-known; from the polyproteins of picornaviruses, flaviviruses and retroviruses [16] to the maturation of receptor-binding proteins in orthomyxoviruses [17]. There are now 10 described SSVs from hot springs throughout the world [2,15]. All known SSVs contain *VP1* genes with N-terminal extensions and conserved C-termini, including the universally conserved glutamate residue [2,15]. (Figure 1). While the C-terminus is extremely well conserved with amino acid identities between 78% and 97% to the SSV1 VP1 sequence, the sequences of the N-termini of SSV VP1s are much less so and their lengths are highly variable (Figure 1). This lack of conservation raises the questions of whether proteolysis is required, the N-terminus of the VP1 protein is required, whether a match between the N-terminus and C-terminus of the protein is necessary, and whether the conserved glutamate residue is required for virus function.

In order to address the role of the N-terminus in VP1 function, we refined the long-inverse PCR technique which we developed to delete ORFs in the SSV1 genome [15,18,19] to make partial deletions, point mutations and complement mutants in the SSV1 genome. Both complete and partial deletions of the N-terminal region of the *vp1* gene of SSV1 led to non-functional virus. These deletions could be complemented with *vp1* genes or portions thereof from homologous SSV *vp1* genes indicating that proteolytic cleavage is necessary for virus function, but proteolysis is not specific to one type of SSV. Changing the conserved glutamate at the putative proteolytic cleavage site did not destroy activity, but virus particles with aberrant morphology were generated.

## 2. Materials and Methods

### 2.1. Strains and Cell Growth

All plasmids (Table 1) derived from EAI283 were grown in *Escherichia coli* strain Transformax EC100D pir^+^ (Epicentre, Madison, WI, USA). All plasmids derived from pAJC97 were grown in NovaBlue cells (Millipore, Burlington, MA, USA). *Sulfolobus* strain S441 was isolated from Lassen Volcanic National Park [8] and was used for *Sulfolobus* transformations and as an indicator lawn for halo assays. *Sulfolobus* cells were grown in Yeast-Sucrose (YS) media [20] and *E. coli* were grown in Lysogeny Broth (LB) with appropriate antibiotics [21,22]. Chemically competent *E. coli* and electrocompetent *Sulfolobus* were prepared and transformed as in Iverson et al. [15].

### 2.2. Purification of Template DNA for Long Inverse PCR from E. coli

*E. coli* cells harboring the pAJC97 or EAI283 SSV1 shuttle vectors (Table 1) were grown overnight in 5 mL of LB medium containing 50 μg/mL Kanamycin. Plasmid DNA was then purified from 1.5 mL of overnight culture via alkaline lysis [23]. Following ethanol precipitation, the DNA was dissolved in 30 μL diH_2_O with 0.01 μg of RNase A. DNA was analyzed by EcoRI restriction endonuclease digestion following the manufacturer’s protocol (New England Biolabs, Ipswich, MA, USA).

### 2.3. Long Inverse PCR 

Long Inverse PCR (LIPCR) was used to delete the *VP1* gene, portions of the *VP1* gene, or to introduce single base pair mutations into the SSV1 genome [15,18,19]. All primers are listed in Table 2. For *VP1* single base pair mutations, primers were designed such that ligation of the LIPCR product resulted only in the desired base pair change. Optimal concentrations of template DNA for LIPCR was determined empirically for each set of primers. LIPCR was performed as described previously [19]. Briefly, LIPCR reactions (20 μL) were prepared following the DNA polymerase manufacturer’s protocol except that the polymerase concentration was reduced from 0.02 U/μL to 0.005 U/μL. Initial annealing temperatures (Ta) for each primer pair were estimated using NEB Tm (melting temperature) prediction software (http://tmcalculator.neb.com/#!/) and were optimized experimentally. The reaction that generated the most full-length product with the least smaller products was selected. LIPCR products were purified, ligated, and transformed into chemically competent NovaBlue (Millipore, Burlington, MA, USA) or Transformax EC100D pir^+^ *E. coli* (Epicentre, Madison, WI, USA) as described previously [19].

### 2.4. Complementation in cis of SSV1 Mutants

SSV1 mutants lacking *VP1* genes or portions thereof were constructed via LIPCR using EAI283 as template (Table 1). SSV2 and SSV9 DNA were isolated from the original *Sulfolobus* strains using alkaline lysis [23]. The complete *VP1* genes from SSV1, SSV2 and SSV9 and the N-terminal region of VP1 from SSV9 [24,25,26] were amplified using Phusion DNA polymerase (New England Biolabs, Ipswich, MA, USA), purified with PCR Purification Kit (Thermo-Fisher, Waltham, MA, USA) and phosphorylated with T4 polynucleotide kinase according to manufacturer’s protocols (Thermo-Fisher, Waltham, MA, USA). DNA containing *VP1* or fragments thereof was heat treated for 10 min at 75 °C prior to ligation reaction. LIPCR products were purified directly from the LIPCR reaction by sodium acetate/ethanol precipitation [21]. The amplified *VP1* gene was mixed with LIPCR product at a molar ratio of 10:1 respectively and ligated with 5 Units of T4 DNA ligase (Thermo-Fisher, Waltham, MA, USA) for 20 h at 16 °C. 5 μL of the ligation reaction was used to transform 100 μL chemically competent Transformax EC100D pir^+^ *E. coli* (Epicentre, Madison, WI, USA).

### 2.5. Transformation of Sulfolobus

*Sulfolobus* was transformed essentially as described by Schleper and colleagues [7]. Cultures of *Sulfolobus* S441 (5 mL) were grown from frozen stocks for 48–72 h in a 70 °C shaking incubator. Starter cultures were transferred to 50–100 mL of fresh YS media in long neck Erlenmeyer flasks and grown until OD_600 nm_ reached ~0.20. Then, 50 mL of cells were removed and placed on ice for 30 min. Cells were washed three times with decreasing volumes of 20 mM ice-cold sucrose as described previously [7]. After the final wash, cells were resuspended in 400 μL ice-cold 20 mM sucrose and kept on ice. Then, 100 μL of cells were added to a chilled 0.1 cm gap length electroporation cuvette (Bulldog Bio, Portsmouth, NH, USA) and 2 μL of SSV or shuttle vector DNA (100–500 ng/μL) purified from *E. coli* using the GeneJET plasmid purification kit (Thermo-Fisher, Waltham, MA, USA) was added to the cells. Cells were transformed by electroporation (BioRad Gene Pulser II, Bio-Rad Laboratories, Hercules, CA, USA) under the following conditions: 1.5 kV, 400 Ω, 25 μF. Immediately following electroporation, cells were resuspended in 1 mL of 70 °C YS, transferred to a 1.5 mL tube, and incubated for 1 h in a 70 °C dry incubator. Following incubation, cells were transferred to 50 mL of preheated YS in a long neck Erlenmeyer flask and grown with shaking at 70 °C.

### 2.6. Halo Assay to Check for Infectious Virus Production

Following electroporation of *Sulfolobus* with SSV DNA, halo assays were performed in duplicate 48 and 72 h post-transformation. Halo assays were performed as previously described [19] by spotting 5 μL of transformed culture on an indicator lawn of *Sulfolobus* strain S441. After 48 h of incubation at 75 °C, a zone of growth inhibition or halo is observed around spots of strains containing infectious virus, since virus production slows growth of the indicator strain. For all halo assays, both a positive control consisting of *Sulfolobus* transformed with an active SSV1 genome and an untransformed negative control were included. Halo assays were only scored if, on the same plate, negative controls did not produce halos and positive controls did. A DNA was determined to be non-infectious after at least 5 independent transformations that did not produce halos.

### 2.7. Confirmation of Infectious SSV DNA

Transformed cultures that produced halos were further analyzed to confirm the identity and purity of the viral DNA. Viral DNA was purified from infected *Sulfolobus* cells using a GeneJET kit (Thermo-Fisher, Waltham, MA, USA). Purified DNA was then amplified using DreamTaq DNA polymerase (Thermo-Fisher, Waltham, MA, USA) using primers that flank the mutated region of the viral DNA. All PCR products were confirmed using DNA sequencing. Control reactions using the DNA used in transformation of *Sulfolobus* and wild type SSV1 DNA were always performed. *Sulfolobus* cultures harboring infectious SSV1 mutants were conserved at −80 °C [20].

### 2.8. Transmission Electron Microscopy

For transmission electron microscopy, samples were prepared on 400-mesh carbon-Formvar-coated copper grids (Ted Pella, Redding, CA, USA). Grids were placed, carbon-Formvar down, on a 5 μL droplet of culture supernatant for 2 min. Culture supernatants were generated by centrifugation at 3000× *g* for 5 min. Samples were removed from the grid by wicking. Grids were then stained for 15–60 s on 5 μL of either 2% uranyl acetate stain (pH 3) or 2% sodium phosphotungstate tribasic hydrate stain (pH 6). Phosphotungstate stain was made freshly every week to ensure that the solution did not disassociate. Grids were allowed to dry in air overnight and were examined within 48 h of staining. Images were obtained at 8500 to 34,000 magnification on an FEI Tecnai F20 transmission electron microscope (TEM) (FEI Inc. Hillsboro, OR, USA). Grids were analyzed by examining randomly selected grid squares. Images were obtained with a BM UltraScan camera and stored in digital micrograph 3 (Gatan, Pleasanton, CA, USA) and TIFF formats.

### 2.9. Particle Analysis

The length and width of images of stained virus particles were measured in Image J [27]. Normal particle width, length, and aspect ratio were determined using the means of measurements of wild type SSV1 particles (*n =* 240) [15]. Any particles whose width, length, or aspect ratio was more than two standard deviations from the means were classified as abnormal. 

## 3. Results

### 3.1. The N-Terminus of the SSV1 Major Capsid Protein VP1 Is Essential for Infectivity

The SSV1 *VP1* gene encodes the major capsid protein [11]. Thus, it is not surprising that a complete deletion of the *VP1* gene and a transposon insertion in the middle of the ORF both failed to yield infectious virus [15]. The VP1 protein appears to be proteolytically cleaved at an internal glutamate residue that is conserved in all known fuselloviruses [15] (Figure 1) to produce the mature protein [5,12]. To investigate if the encoded N-terminus is required for infectivity, this region was deleted using LIPCR while leaving the conserved glutamate intact and maintaining a start (ATG) codon (Figure 1). The construct was electroporated into *Sulfolobus* and infectivity tested by halo assay. Infectious virus was repeatedly not produced by this *VP1*-∆N-terminus mutant (EAI564), suggesting that the VP1 N-terminus is required for production of infectious virus. The N-termini of fusellovirus VP1 proteins are much less well-conserved than the C-termini, with the exception of a group of well-conserved residues just N-terminal to the conserved glutamate (Figure 1). This region was deleted from the SSV1 *VP1* gene and the resulting mutant (EAI578) was also found to be non-infectious. Thus, the N-terminus of VP1 and 5 amino acids immediately N-terminal to the cleavage site appear to be essential for infectivity.

### 3.2. Complementation in cis of SSV1 Deletion Mutants

**Figure 2:**
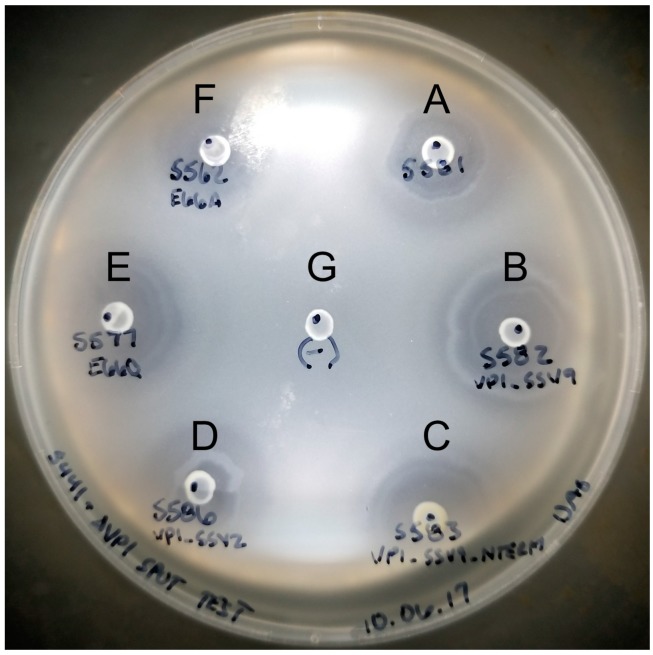
Spot on lawn “halo” assays for functional SSV1 *VP1* mutants. *Sulfolobus* cultures infected with SSV1 containing various *VP1* mutations were spotted on a lawn of uninfected *Sulfolobus*. The lawn and infected cultures were allowed to grow for 48 hours and then photographed. Letters indicate indicates a spotted culture of *Sulfolobus* infected with a SSV1 that contains the wild type *VP1* gene (**A**); the *VP1* gene from SSV9 (**B**); a fusion between the N-terminus of VP1 from SSV9 and the C-terminus of VP1 from SSV1 (**C**) the *VP1* gene from SSV2 (**D**); a glutamine at position 66 in the SSV1 VP1 protein instead of the wild type glutamate (**E**), or an alanine at position 66 in the SSV1 VP1 protein instead of the wild type glutamate (**F**); (**G**) indicates an uninfected control culture spotted on the lawn.

In order to confirm that SSV1 *VP1* deletions were non-functional only due to the deletion, we complemented the mutants *in cis* with the SSV1 wild type *VP1* gene and with homologs from other SSVs. Infectivity of the SSV1 ∆*VP1* mutant could be rescued when complemented *in cis* with the SSV1 wild type *VP1* gene, or homologous *VP1* genes from either SSV2 or SSV9 (Figure 2A–D). The infectivity of the non-functional *VP1*-∆N-terminus mutant (EAI564) was also rescuable by complementation *in cis* when the VP1-N-terminus from SSV9 was added to the C-terminus of SSV1 VP1 (Figure 2C). The C-termini of the three VP1 proteins are highly similar; however, the N-terminus of SSV2-VP1 is considerably shorter (15 residues) than the other two (SSV9-VP1 65 residues and SSV1-VP1 66 residues, respectively) (Figure 1). Virions with these alternative *VP1* genes had normal morphology on analysis with TEM.

### 3.3. Changing the Conserved Glutamate in the Major Capsid Gene VP1 Allows Infectious Virus but Generates Many Abnormal Virions

**Figure 3:**
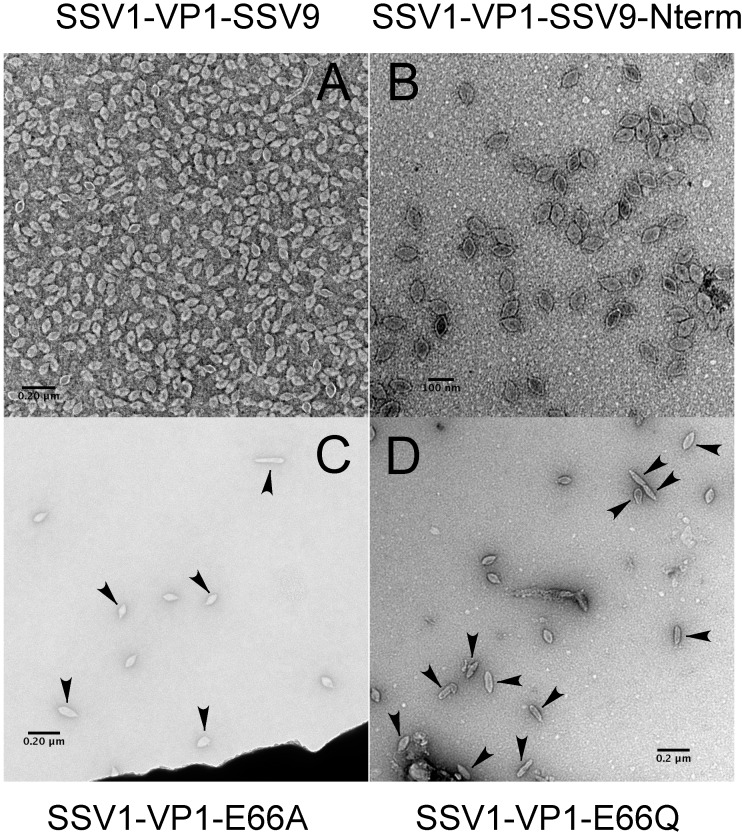
Transmission electron micrographs of SSV1 *VP1* mutants. Negatively stained SSV1 mutant virions were imaged using a Tecnai F-20 (FEI) transmission electron microscope operating at 200 keV. The culture supernatant in (**Panel A**) was concentrated ca. 100 fold with a Vivapsin-500 (10 kDa cutoff) concentrator. (**Panel A**) Virions from SSV-1 containing the *VP1* gene from SSV9; (**Panel B**) Virions from SSV-1 containing a *VP1* gene with the N-terminus of the gene from SSV9; (**Panel C**) Virions from SSV-1 containing a *VP1* gene with the E66A substitution; (**Panel D**) Virions from SSV-1 containing a *VP1* gene with the E66Q substitution. Arrowheads in (**Panels C,D**) indicate “abnormal” particles.

Although the presence of a N-terminus of VP1 is essential, the necessity for proteolysis at glutamate 66 for the activity of SSV1 remained unclear. Thus we investigated whether the universally conserved glutamate of VP1 itself is essential for infectivity. A single base pair change was made in the *VP1* gene by LIPCR, converting the glutamate to the structurally similar but functionally distinct glutamine (EAI427; Table 1). Interestingly, viruses containing this mutation were infectious as assessed by halo assay (Figure 2E). Even when the glutamate was converted to alanine (EAI500), the virus was still infectious (Figure 2F). However, when analyzed by TEM, virions containing these point mutations had a very high proportion of abnormally shaped virions (Figure 3). Those viruses that had the E66Q substitution had 57% (*n* = 198) abnormal morphology and those with the E66A 81% (*n* = 99). Wild type SSV1 preparations have 5.4% abnormal particles (*n* = 240) [15]. Thus, glutamate residue 66 is not absolutely essential for SSV1 infectivity despite its universal conservation in the Fuselloviridae.

## 4. Discussion

### 4.1. The Translational Start of the VP1 Protein Is Not Clear

Here we show that the N-terminus of the SSV1 VP1 protein is required for infectivity. Since the VP1 protein in intact SSV1 virions has a glutamate at its N-terminus, the identity of the actual start codon that is used to translate the SSV1 VP1 precursor protein is not clear [5]. Based on sequence comparison (Figure 1), the presumed start codon is a TTG. ATG, TTG and GTG codons are known to be used as start codons for archaeal translation [28] but there is an in-frame ATG codon 18 nucleotides upstream of this TTG that is 9 nucleotides downstream of the transcriptional start site, a normal distance for translational initiation [29]. However, there is no clear *Sulfolobus* ribosome binding site at any of these potential translational start sites [30]. Thus the SSV1 *VP1* gene may be misannotated. However, there is a conserved open reading frame, SSV1 ORF b115, that overlaps the first 12 codons of the predicted SSV1-*VP1* gene [5]. Interestingly, there is a similar 14 codon overlap in the SSV2 genome even though the predicted N-terminus of the SSV2 *VP1* gene is much shorter than that of SSV1 [26]. Whether these overlapping ORFs are translated, however, is not known. This confusion regarding the SSV1-VP1 precursor protein makes analysis of the protease and proteolysis challenging, as it is not clear what the appropriate substrate is. A replacement of the N-terminus of SSV1 with the N-terminus from SSV2 could be a useful tool to study this proteolytic process and the N-terminus of the preprotein would be better defined.

### 4.2. The N-Terminus of SSV1 VP1 Is Essential, But the Cleavage Site Is Not

When the entire N-terminus of SSV1 VP1 is deleted, or 5 amino acids N-terminus to the cleavage site are deleted, no viable virus was produced (Figure 1). This indicates that cleavage of the VP1-precursor is necessary and depends on the 5 amino acids next to the cleavage site. However, when a point mutation was introduced changing the conserved cleavage site glutamate (E) to either a glutamine (Q) or alanine (A) (E66Q and E66A), infectious virus particles were produced, indicating that some cleavage is taking place. It is unknown if VP1 proteolysis can still occur at this position in either of the mutants or if proteolysis occurs at one of the other well-conserved glutamate residues upstream (Figure 1). Experiments to address the site of proteolysis are underway, but yields of SSV virus preparations are notoriously low [7,11,12]. Comparative stability experiments with these active mutants are also being performed.

### 4.3. Virions Containing Point Mutations in VP1 Have Unusual Morphology

We previously showed that deletions of the gene for the minor capsid protein, *VP3* in SSV1, led to the production of longer and thinner virions. Fully 99% of these mutant viruses were abnormal, as defined by deviating from means of length, width and aspect ratio of the wild type virus by more than 2 standard deviations [15]. Unlike VP3 deletions, virions with a mutated VP1 cleavage site had some normal and some abnormal morphologies. Often the abnormal particles had differently shaped ends, some pear-shaped. Interestingly there were fewer abnormal (57%) virions containing the E66Q mutation than those containing the E66A mutation (81%). This indicates that there may be some proteolysis at amino acid 66 and more proteolysis for the structurally similar amino acid Q than for the structurally dissimilar amino acid A.

### 4.4. The SSV1 VP1 N-Terminus Can Be Functionally Replaced with the SSV9 VP1 N-Terminus

Even though cleavage of the N-terminus appears to be essential for al SSVs, the lack of conservation of the N-termini led us to suspect that the amino acid sequence of the N-terminus is not critical. To confirm this, we switched the N-terminus of SSV1’s VP1 with the N-terminus of SSV9 VP1 [25] (Figure 1). Whether this can be done with the predicted N-terminus of the SSV2 VP1 protein that is much shorter than that of SSV1, remains to be determined.

### 4.5. Proteolysis and Assembly of SSV1

Proteolytic processing during virion assembly is common and may be the rule rather than the exception [31]. We have demonstrated that the N-terminus of VP1 SSV1 is essential for infectivity. The N-termini of fusellovirus *VP1* genes are not well conserved with the exception of the essential patch of residues just upstream of the conserved glutamate (Figure 1). Complementation of ∆*VP1* mutants with SSV2-*VP1*, which possesses a truncated N-terminus, suggests that the majority of the VP1 N-terminus can be dispensed with as long as the residues upstream of the conserved glutamate remain intact. The role of these essential residues remains mysterious. These residues might be critical for recruitment of a protease and/or may act as a scaffold, potentially via interactions with neighboring VP1 N-termini, the minor capsid protein VP3, or some other protein involved in assembly. The identity of proteases or scaffolding proteins for SSV1 is not known. The protease could be viral, cellular or even the VP1 protein itself. There are no ORFs in the SSV1 genome that have clear similarity to proteases, but there are a few known proteases in the *Sulfolobus* genome [32,33]. However, none of these have specificity for glutamate. Assembly is likely highly conserved among the Fuselloviridae and any protein scaffolding candidate would likely come from the set of core fusellovirus genes [15]. The hypothesis that the N-terminus of VP1 may fulfill this role is an intriguing one and awaits further research.

## 5. Conclusions

We show that the N-terminus of the SSV1 major capsid protein VP1 is essential for virus function, probably for assembly and proteolysis. Similar to many other viruses, proteolysis itself appears to be essential to allow the capsid proteins to form the spindle-shaped morphology. The protease that cleaves the N-terminus is unknown but may have some activity on non-conserved amino acids. The presence of a point mutation in the cleavage site leads to the production of aberrant virus particles, probably containing N-terminal extensions on VP1. The N-terminus of VP1, in addition to providing a site for proteolysis may also provide a scaffolding role. Future research will concentrate on the identification of the novel protease and role of individual amino acids in the N-terminus of SSV1 VP1 in virus assembly.

## Figures and Tables

**Table 1:** Plasmids used in this study.

Plasmid	Description	Infectivity	Reference
pAJC97	SSV1 shuttle vector (TOPO PCR Blunt II inserted into ORF ^A^ *e178* at bp 3173)	+	[18]
EAI283	SSV1::Tn5 mutant, EZ-Tn5 inserted at bp 3572 (ORF *e178*)	+	[15]
EAI427	pAJC97 background with E66Q mutation in VP1	+	This Work
EAI500	pAJC97 background with E66A mutation in VP1	+	This Work
EAI551	EAI283 background with SSV1-*VP1* complementation	+	This work
EAI566	EAI283 background with SSV2-*VP1* complementation	+	This Work
EAI553	EAI283 background with SSV9-*VP1* complementation	+	This Work
EAI557	EAI283 background with N-terminus of SSV9-VP1 complementation	+	This Work
EAI237	pAJC97 background with N-terminus of VP1 deleted	−	This Work
EAI564	EAI283 background with N-terminus of VP1 deleted	−	This Work
EAI578	EAI283 background with residues 61–65 deleted from VP1	−	This Work

^A^ Open Reading Frame (ORF).

**Table 2:** Primers used in this study.

Product	Forward Primer	Reverse Primer
SSV1 *VP1* deletion	**TGA** GGG ATG GAA ATC AGT TTA AAG	**CAA** ACT CCT TAG GAG TCT CAT CC
SSV1 VP1 N-terminus deletion	*ATG* **GAA GCA ACC AAC ATA GG** (61) and **GAA GCA ACC AAC ATA GG** (54)	**CAA** ACT CCT TAG GAG TCT CAT CC
SSV1 VP1 aa61–65 deletion	**GAA GCA ACC AAC ATA GGC**	**GGG GTT TGC CTT TGC TAC**
SSV1 VP1 point mutant (E66A) ^A^	**GCA GCA ACC AAC ATA GGC**	**ACC TTT TGT GAG CTT GGG G**
SSV1 VP1 point mutant (E66Q) ^B^	**CAA GCA ACC AAC ATA GGC**	**ACC TTT TGT GAG CTT GGG G**
SSV1 *VP1* ^C^	GCcAGAAAGATAGCCTCAC	ACCTTTTGTGAGCTTGGG
SSV9 *VP1*	GAAGTTTGGTCAAAGTTAAACG	ATCTTTGTAGATTTTATACG
SSV2 *VP1*	GCCACCAGACTAATGCTAAGC	GTCACGATATATCTTATACGCTATGAC
SSV9 VP1 N-terminus	GAAGTTTGGTCAAAGTTAAACG	ACCCCTAGTAAGTTTGGG

All sequences are written 5′ → 3′, bases in bold type are within the SSV1 ORF; ^A^ Highlighted base indicates mismatch generating Glu→ Ala mutation in VP1 protein; ^B^ Highlighted base indicates mismatch generating Glu→ Gln mutation in VP1 protein; C Lower case denotes introduced silent restriction endonuclease site. *ATG:* start codon.
